# Chest pain and eosinophilia in a middle-aged female caused by hookworm infection: a case report

**DOI:** 10.1590/S1678-9946202668052

**Published:** 2026-08-07

**Authors:** Bo Jiang, Xiaoyan Zhu

**Affiliations:** 1 The First Affiliated Hospital of Kunming Medical University, Department of Clinical Laboratory, Kunming, China; 2 Yunnan Key Laboratory of Laboratory Medicine, Kunming, China; 3 Yunnan Province Clinical Research Center for Laboratory Medicine, Kunming, China

**Keywords:** Hookworm infection, Chest pain, Eosinophilia, Stool microscopy.

## Abstract

Hookworm infection, while often asymptomatic, may rarely cause Löffler syndrome. We report the case of a 45-year-old female farmer who presented with a 10-year history of recurrent chest pain. Laboratory tests revealed marked eosinophilia (33.4%) and microcytic anemia. Cardiac and abdominal imaging findings were normal. Stool microscopy confirmed hookworm infection. Treatment of oral mebendazole (400 mg twice daily) for three days resulted in complete resolution of symptoms, normalization of eosinophil count, and clearance of parasites on follow-up. This case highlights hookworm infection as a potential cause of eosinophilia and chest pain in endemic regions.

## INTRODUCTION

Hookworms are intestinal parasites that pose a major public health problem, particularly in developing countries. According to the World Health Organization (WHO), approximately half a billion individuals are infected with hookworms globally, mainly in tropical and subtropical regions, including Southeast Asia, Papua New Guinea, most of Africa, and Central and South America ^(^ ^1^. Hookworm infection has also been one of the most common soil-transmitted helminthiases in China. Most infected individuals are asymptomatic, although a minority develop anemia and malnutrition ^(^ ^2^. In rare cases, hookworm infection may lead to Löffler syndrome, which is characterized by severe cough, wheezing, eosinophilia, hemoptysis, and other signals and symptoms ^(^ ^3^.

The diagnosis of hookworm infection is straightforward and can be performed using basic laboratory facilities, even in low-income countries. Traditionally, diagnosis relies on microscopic examination of stool samples for hookworm eggs. However, because egg excretion may be intermittent, this method has limited sensitivity and is highly dependent on the expertise of the microscopist. Nevertheless, stool microscopy remains a highly valuable diagnostic tool for suspected parasitic infections because it is widely available, cost-effective, and capable of detecting a range of intestinal parasites. Studies have demonstrated that, in high-prevalence areas, microscopic examination can effectively identify multiple intestinal parasites and, in some cases, detect infections that may be missed by certain molecular diagnostic methods. A study conducted in South Korea, for example, found that routine stool parasitology tests detected a significant number of occult worm infections, underscoring the importance of microscopic examination in health screenings^4^. Therefore, stool microscopy should remain a first-line diagnostic approach in patients with suspected parasitic infections. Here, we report the case of a 45-year-old woman presenting with chest pain and eosinophilia caused by hookworm infection. Additionally, we briefly review the literature on hookworm infection to discuss the key clinical findings, diagnosis, and treatment strategies.

## ETHICS

Ethical approval was granted by the Ethics Committee of First Affiliated Hospital of Kunming Medical University (process No. 2021-L-20).

## CASE REPORT

A 45-year-old woman presented to the cardiology clinic with a 10-year history of recurrent chest tightness and chest pain, accompanied by headache, nausea, and palpitations. The episodes usually lasted for 10 min and resolved spontaneously. She had smoked three to four cigarettes per day for 20 years and had no significant medical history except for the aforementioned symptoms.

**Figure 1:**
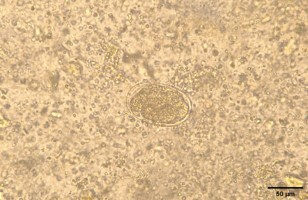
Microscopic identification of a hookworm egg. Stool microscopy revealed a characteristic hookworm egg with an oval shape, a thin, translucent shell, and internal blastomeres.

At presentation, the patient reported that she was a rice farmer from Xishuangbanna, Yunnan, China. Vital signs showed a blood pressure of 121/82 mm Hg, a heart rate of 57 beats/min, a respiratory rate of 20 breaths/min, an oxygen saturation of 95%, and a temperature of 36.6 °C. Laboratory tests revealed a white blood cell count of 9,760/μL (reference range, 3,500-9,500/μL), with eosinophils accounting for 33.40% (reference range, 0.40%-8.0%) (3,260/μl). Platelet count was 394,000/μL, and hemoglobin level was 11.1 g/dL (reference range, 11.5-15.0 g/dL for women). A peripheral smear showed microcytic, hypochromic red blood cells (RBCs). Blood glucose, electrolyte, coagulation, renal function, and liver function tests were all within normal laboratory limits. The patient was admitted to the hospital due to chest pain. Coronary computed tomography angiography (CTA), echocardiography, and abdominal ultrasound were normal. The stool appeared normal, without blood or melena, although the fecal occult blood test was positive. A direct saline wet mount of the stool sample was examined under light microscopy, revealing hookworm eggs (Figure 1), which were identified according to standard morphological criteria^5^.

She subsequently completed a three-day course of oral mebendazole (400 mg twice daily), which was well tolerated without complications. She was discharged in good clinical condition on the third day after treatment. Before discharge, the patient received detailed instructions on preventing reinfection, including wearing shoes while walking on soil, washing her hands after soil contact, thoroughly cooking vegetables, and avoiding open defecation near the household. She confirmed her understanding and agreed to follow these recommendations. One month later, her clinical symptoms were greatly improved. Eosinophil levels were normal, repeat stool microscopic examination was negative for hookworm eggs, and serum ferritin levels had normalized.

## DISCUSSION

Hookworm infections are among the most common neglected tropical diseases worldwide. Human hookworm infections are primarily caused by *Necator americanus*, *Ancylostoma duodenale*, and *Ancylostoma ceylanicum*, which are distributed throughout sub-Saharan Africa, the Americas, and Asia^6^. In China, hookworm infection is most prevalent in southern regions, particularly in the Southwest (excluding Tibet), South, and Southeast, with Sichuan, Yunnan, Chongqing, and Hainan being the most affected provinces^7^. Recent studies have shown a decline in infection rates after improvements in sanitation and public health measures; however, hookworm infection remains a significant public health concern, especially in rural areas^8^ ^),(^ ^9^.

The pathogenic mechanisms of hookworm infection differ between the larval and adult stages. Adult hookworms attach to the small intestinal mucosa, ingest blood, and secrete anticoagulants that exacerbate bleeding at the attachment sites^10^. Over time, cumulative iron loss leads to microcytic hypochromic anemia, as observed in this case. Disease severity depends on worm burden, infection duration, and host iron reserves. In endemic areas such as Xishuangbanna, even recurrent low-intensity infections may contribute to progressive iron depletion. In contrast, migrating larvae trigger an immune response during tissue migration, resulting in eosinophilia^11^. In this case, the patient had experienced recurrent chest tightness for a long time. When her symptoms worsened and she sought medical attention, eosinophilia became apparent. Because she resided in Xishuangbanna, an endemic region for hookworm infection^12^, recurrent low-intensity infections were considered likely. We propose that pulmonary larval migration, which resulted in localized inflammation and hemorrhage, exacerbated respiratory symptoms in a patient whose respiratory system had already been compromised by chronic smoking and bronchial hyperresponsiveness.

This case highlights that uncommon symptoms such as this require careful medical evaluation and should not be overlooked. In endemic areas, peripheral eosinophilia should raise suspicion of parasitic infection. Moreover, recent studies have shown that intestinal parasitosis, including *Ascaris lumbricoides*, *Necator americanus*, and *Ancylostoma* spp., may cause pulmonary eosinophilia (Löffler syndrome) during migration through lung tissue, presenting with chest tightness, cough, and associated marked blood eosinophilia. Crucially, hookworm infection may occur in the absence of classic gastrointestinal symptoms, such as abdominal pain and diarrhea, rendering respiratory symptoms accompanied by eosinophilia pivotal early diagnostic clues. Consequently, in patients presenting with unexplained chest tightness and eosinophilia, particularly middle-aged and older adults with potential exposure risks, parasitic infections, including hookworm infection, must be rigorously included in the differential diagnosis to prevent misdiagnosis and therapeutic delay.

Laboratory diagnosis of hookworm infection relies on the detection of ova or larvae in stool samples, which can be influenced by the amount of egg or larval shedding. It has been reported that a minimum of 48 eggs per gram of feces and the detection of eggs on both of two slides are required for confirmed diagnosis, while false negatives are more likely to occur under low-intensity infection^13^. Furthermore, although the eggs of *Ancylostoma duodenale* and *Necator americanus* are virtually indistinguishable by light microscopy, molecular methods offer improved sensitivity and specificity, while enabling species differentiation. Despite the availability of more sensitive molecular tests, conventional stool microscopy remains the primary parasitological examination for diagnosing hookworm infection, especially in low-resource endemic communities. Benzimidazoles are currently the first-line treatment for hookworm infection. Recommended treatment regimens include a single 400 mg dose of albendazole, a single 500 mg dose of mebendazole, or mebendazole 100 mg twice daily for three days^14^. Based on a previous study showing that a single 400 mg dose of albendazole achieved a cure rate of only 65.1% against hookworm infection, the patient in this case received mebendazole at a dose of 400 mg twice daily^15^. Albendazole often requires co-administration with other drugs, such as ivermectin, to improve efficacy, which can increase the risk of adverse drug reactions^16^. Furthermore, mebendazole has low water solubility and high lipophilicity, properties that may promote maintenance of higher concentrations in adipose tissues or specific organs, such as the intestinal tract, thereby potentially enhancing its therapeutic effect^17^. Treatment efficacy may vary according to hookworm species and patient age. Different hookworm species exhibit distinct responses to anthelmintic drugs. For instance, a previous study revealed a mean cure rate of 91.8% for *Ancylostoma duodenale* and 75.0% for *Necator americanus* following albendazole treatment^18^. However, another study found that a single dose of albendazole achieved a cure rate of only 36.0% for duodenal hookworms, while demonstrating higher efficacy against *Ancylostoma caninum* ^19^. Additionally, patient age may influence treatment efficacy. Studies indicate that younger patients experience greater reduction in parasite eggs after albendazole treatment, which may be related to their higher metabolic rate and immune response^20^. Therefore, the efficacy of hookworm treatment may vary depending on both parasite species and patient characteristics. Future studies should further investigate how these factors influence treatment outcomes to support the development of more precise therapeutic strategies.

## CONCLUSION

This case of chest pain and eosinophilia in a middle-aged female due to hookworm infection underscores the importance of considering helminthic infections in the differential diagnosis of atypical presentations. Prompt treatment with mebendazole resulted in complete clinical recovery. This case also emphasizes the ongoing public health challenges posed by hookworm infections, particularly in endemic regions such as Yunnan.

## Data Availability

The Complete Anonymized Dataset Supporting The Findings Of This Study Is Included Within The Article Itself.
